# Supplementary material for: MetaRibo-Seq measures translation in microbiomes
Source: Nat Commun. 2020 Jun 29;11:3268. doi: 10.1038/s41467-020-17081-z (PMC7324362; doi:10.1038/s41467-020-17081-z)
Supplement: Supplementary file 10 — Supplementary Data 7 [file 41467_2020_17081_MOESM10_ESM.zip › File2/Confidence_High_Taxonomy/155473_out.krona.html]

Javascript must be enabled to view this page.

members
magnitude
magnitudeUnassigned
count
unassigned
taxon
rank

155473\_out

4

4
2
superkingdom

2
976
phylum

117743
2
class

order
200644
2

family
2
49546

genus
2
1016

species

SRS011126\_contig\_number\_15535SRS064449\_contig\_number\_26720
2
1017

phylum
2
1239

186801
2
class

order
2
186802

2
541000
family

genus
1
292632

SRS143876\_contig\_number\_8432
2

species
1

SRS149244\_contig\_number\_7415
665956
